# Sleep duration and chronotype of pregnant women in the United States: An online cross-sectional survey study

**DOI:** 10.1016/j.pmedr.2022.102088

**Published:** 2022-12-06

**Authors:** Elizabeth M. Cassidy, Caitlin P. Bailey, Melissa A. Napolitano, Amita N. Vyas

**Affiliations:** Milken Institute School of Public Health, The George Washington University, Washington, D.C., USA

**Keywords:** Pregnancy, Chronotype, Sleep duration, Maternal-child health

## Abstract

Sleep is an important behavior, and during pregnancy plays a critical role in promoting the health of both woman and child. Therefore, identifying and addressing sleep parameters during pregnancy, and associated disparities in maternal-child health outcomes, is a public health priority. This studied aimed to examine chronotype and sleep duration in pregnant women by sociodemographic factors. An online survey was distributed to currently pregnant women living in the greater Washington, DC, area of the United States from March to May of 2022. The survey included the ultra-short version of the Munich Chronotype Questionnaire (µMCTQ), which was used to calculate the mid-point of sleep time (MST; a measure of chronotype) and sleep duration, as well as demographic questions. Linear regression was used to assess differences in sleep outcomes by demographic factors. A sample of n = 142 currently pregnant women were eligible for analysis. In covariate adjusted models, sleep duration was longer among respondents ages 25–34 (09:52) compared with younger respondents ages 18–24 (08:59; p = 0.014), and shorter for those with a household income ≥ 100,000 United States dollars (USD) (07:42) compared with those with a household income < 50,000 USD (08:59; p = 0.001). No differences in mean mid-point of sleep were identified across demographic factors. Differences in sleep duration by age and household income were found among currently pregnant women. Future studies should investigate sleep parameters and other factors that may influence maternal-child health disparities.

## Highlights

• Sleep duration was longer among those ages 25–34 versus 18–24.
• Sleep duration was shorter for those earning ≥ 100,000 USD versus < 50,000 USD.
• No differences in chronotype were identified across demographic factors.

## 1 Background

Sleep is an important health behavior throughout the lifecycle (Ross, 2020, Tremblay, 2016); for example, it plays a significant role in the health of both mother and child during pregnancy (Chang, 2010). Despite the importance of sleep during pregnancy, guidelines for sleep duration specific to pregnant women currently do not exist. Generally, poor sleep, which can be measured using a variety of dimensions (e.g., onset, duration, continuity) is associated with negative maternal-child outcomes, such as perinatal depression, prolonged labor leading to cesarian section, and premature birth (Tsai, 2011). Furthermore, chronotype – defined as the preference for timing of sleep onset and waking – may be predictive of increased risk for maternal complications throughout pregnancy, labor, and delivery (Feinstein, 2020). Given the alarming disparities in maternal-child health in the United States (Petersen, 2019), coupled with growing evidence of the importance of sleep hygiene for health (Ross, 2020, Tremblay, 2016), understanding the potential impact of sleep parameters on maternal-child health outcomes is a public health priority.

Previous research has found that both sleep duration and chronotype significantly differ among pregnant versus non-pregnant women. Pregnant women have later sleep onsets, more night waking, and shorter total sleep duration, particularly in the third trimester (Mindell et al., 2015), indicating that knowledge of sleep duration and chronotype prior to pregnancy may not be enough to predict pregnancy-related health outcomes. Data regarding sleep parameters *during* pregnancy are key to understanding potential predictors of negative maternal-child health and birth outcomes given that women experience unique physiological and psychological experiences during this time. Pain, discomfort, reflux, and frequent urination are the most commonly reported physiological reasons for later sleep onset and earlier wake times, while vivid dreams and worry about labor or the baby are the most commonly reported psychological reasons (Mindell et al., 2015).

Other studies examining social determinants of health, particularly financial factors have found that women with an annual household income ≤ $50,000 have reported significantly poorer continuity of sleep and an overall shorter sleep duration compared to women with an annual household income of > $50,000 (Okun et al., 2014). The reason for this association is unknown, though it is hypothesized that other factors related to financial status (e.g., education, employment) may also play a role in sleep outcomes and, ultimately, overall health. Additionally, race has also been identified as a predictor of sleep parameters among pregnant women. Compared to pregnant White women, pregnant Black/African American women are more likely to have short sleep durations (Feinstein, 2020). Given that Black/African American women are three to four times more likely to experience pregnancy-related complications than their White counterparts (Louis et al., 2015), differences in sleep duration and chronotype may be one contributing factor to the disparity in perinatal complications among Black/African American mothers (Feinstein, 2020).

The present pilot study was conceptualized to examine associations between sociodemographic factors (e.g., race, age, education, annual household income) and sleep parameters (i.e., sleep duration, chronotype).

## 2 Methods

### 2.1 Design and data collection

A cross-sectional online survey, built using Qualtrics^XM^ software, was conducted in Spring 2022. Currently pregnant women over 18 years of age living in the greater Washington, DC, area of the United States were eligible to participate in the survey. Participants were recruited via DC-based prenatal clinic email listserves, social media (i.e., Facebook, Instagram, GroupMe), and Centiment (Centiment, xxxx), a web-based survey panel recruitment company. Study procedures were approved by the first author’s institutional review board. A total of N = 282 currently pregnant respondents participated in the survey. Exclusion criteria for the present analysis included: missing responses to any sociodemographic or sleep questions and having been a shift-worker in the past three months. Of the N = 282 currently pregnant survey respondents, n = 62 had been shift-workers in the past three months, n = 67 were missing µMCTQ responses, and N = 11 were missing demographic responses. Therefore, the final analytic sample size was N = 142.

### 2.2 Measures

***Sociodemographic.*** The survey contained questions asking participants to identify their age, race, marital status, education level, annual household income, and employment status.

***Sleep Parameters.*** The ultra-short version of the Munich Chronotype Questionnaire (µMCTQ) was used to assess sleep duration and chronotype, operationalized as the mid-point of sleep onset and wake time, or mid-point of sleep time (MST) (Ghotbi, 2020). Both sleep duration and MST were adjusted for differences in workdays versus work free days by taking a weighted average of the two values, where workdays multiplied by 5 plus work free days multiplied by 2 were divided by 7 (Roenneberg et al., 2003, Suh, 2017).

### 2.3 Analysis

Descriptive statistics were generated for all sociodemographic variables using Fisher’s exact tests (due to cell counts < 5). Unadjusted simple regression models predicting sleep duration and MST from each demographic variable were tested. Finally adjusted regression models predicting sleep duration and MST with all demographic variables entered simultaneously were tested. Partial F-test p-values were generated for variables with more than two levels. Statistical analyses were conducted using RStudio Version 1.3.1056 (Team, 2020). A significance level of < 0.05 was determined a priori.

## 3 Results

### 3.1 Demographics

Descriptive characteristics of the analytic sample are shown in Table 1. The sample was 15 % Black/African American, 40 % White/Caucasian, 21 % Asian/Pacific Islander, and 24 % other racial/ethnic group (i.e., Hispanic/Latino [12 %], Native America/Alaskan Native [4 %], Biracial/Multiracial [4 %], unknown [4 %]). The sample was predominantly between the ages of 25–34, married or in a domestic partnership, holding a Bachelor’s degree, with a household income ≥ 100,000 USD, and employed full-time. Differences in sociodemographic factors by race/ethnicity were identified for education level (p = 0.005) and annual household income (p < 0.001).

**Table 1:** Descriptive characteristics of the analytic sample.

	Full Sample (N = 142)	Black/AA (N = 21)	White (N = 57)	Asian/Pacific Islander (N = 30)	Other† (N = 34)	P-value
Age						0.706
18–24	29 (20.42)	7 (33.33)	10 (17.54)	4 (13.33)	8 (23.53)	
25–34	96 (67.61)	12 (57.14)	39 (68.42)	23 (76.67)	22 (64.71)	
35–44	17 (11.97)	2 (9.52)	8 (14.04)	3 (10.00)	4 (11.76)	
Marital status						0.094
Married/Dom. Partnership	129 (90.85)	18 (85.71)	51 (89.47)	26 (86.67)	34 (100.00)	
Single/Divorced/Widowed	13 (9.15)	3 (14.29)	6 (10.53)	4 (13.33)	0 (0.00)	
Highest Education						0.005**
High school equiv. or less	32 (22.54)	8 (38.10)	9 (15.79)	4 (13.33)	11 (32.35)	
Assoc. degree or trade sch.	18 (12.68)	3 (14.29)	4 (7.02)	5 (16.67)	6 (17.65)	
Bachelor’s degree	52 (36.62)	4 (19.05)	26 (45.61)	10 (33.33)	12 (35.29)	
Master’s degree	27 (19.01)	2 (9.52)	17 (29.82)	5 (16.67)	3 (8.82)	
Professional degree or PhD	13 (9.15)	4 (19.05)	1 (1.75)	6 (20.00)	2 (5.88)	
Annual household income						<0.001**
<50,000	42 (29.58)	10 (47.62)	8 (14.04)	13 (43.33)	11 (32.35)	
$50,000-$99,999	41 (28.87)	7 (33.33)	17 (29.82)	4 (13.33)	13 (38.24)	
≥100,000	59 (41.55)	4 (19.05)	32 (56.14)	13 (43.33)	10 (29.41)	
Employment						0.075
Full-time	75 (52.82)	9 (42.86)	33 (57.89)	21 (70.00)	12 (35.29)	
Part-time	31 (21.83)	4 (19.05)	11 (19.30)	6 (20.00)	10 (29.41)	
Unemployed	36 (25.35)	8 (38.10)	13 (22.81)	3 (10.00)	12 (35.29)	

Note: Statistical comparisons conducted using Fisher’s Exact tests; Bold/italicized p-values are significant; Significance level denoted by *<0.05, **<0.01, ***<0.001; †Other category included Hispanic/Latino, Native America/Alaskan Native, biracial/multiracial, and unknown; AA = African American.

### 3.2 Sleep duration

In unadjusted models, race and annual household income were each associated with sleep duration. Black/African American respondents reported almost one hour more of sleep compared with White respondents (09:06 vs 08:14, p = 0.038). Respondents with household incomes ≥ 100,000 USD reported almost one hour less of sleep compared with those with incomes < 50,000 USD (08:06 vs 09:05, p = 0.003).

In adjusted models where all covariates were entered into the model simultaneously, age and annual household income were significant predictors of sleep duration. Respondents between the ages of 25–34 had almost one hour more of sleep compared with respondents ages 18–24 (09:52 vs 08:59, p = 0.014), and respondents with household incomes of ≥ 100,000 USD had over one hour less of sleep compared with those with incomes < 50,000 USD (07:42 vs 08:59, p = 0.001). Partial F-tests for age and annual household income was significant (ps < 0.046), indicating that after considering the other variables in the model age and annual household income each contributed information to sleep duration. See Table 2.

**Table 2:** Linear regression predicting sleep duration (Model 1) and chronotype, i.e., midpoint of sleep (MST; Model 2), from demographic variables.

	Model 1^a^			Model 2^b^		
	β (95 % CI)	Mean sleep duration	P-value	β (95 % CI)	Mean MST	P-value
Race			0.482†			0.165†
Black/AA	–	08:59	–	–	03:15	–
White	−0.57 (-1.40, 0.26)	08:25	0.177	−0.53 (-1.20, 0.15)	02:43	0.125
Asian/Pacific Islander	−0.30 (-1.20, 0.60)	08:41	0.515	−0.35 (-1.09, 0.38)	02:54	0.343
Other	−0.60 (-1.47, 0.27)	08:23	0.177	0.08 (-0.64, 0.79)	03:20	0.832
Age			0.046*†			0.516†
18–24	–	08:59	–	–	03:15	–
25–34	0.89 (0.18, 1.61)	09:52	0.014*	−0.26 (-0.84, 0.32)	02:59	0.377
35–44	0.92 (-0.11, 1.95)	09:54	0.078	−0.47 (-1.31, 0.36)	02:47	0.265
Marital status						
Married/Dom. Partnership	–	08:59	–	–	03:15	–
Single/Divorced/Widowed	−0.32 (-1.32, 0.67)	08:40	0.519	0.53 (-0.28, 1.34)	03:47	0.196
Annual household income			0.005**†			0.747†
<50,000	–	08:59	–	–	03:15	–
$50,000-$99,999	−0.61 (-1.35, 0.12)	08:22	0.100	−0.02 (-0.62, 0.58)	03:14	0.940
≥100,000	−1.28 (-2.04, −0.51)	07:42	0.001**	0.18 (-0.44, 0.80)	03:26	0.572

Note: Bold/italicized p-values are significant; Significance level denoted by *<0.05, **<0.01, ***<0.001; ^a^Adjusted linear regression predicting sleep duration; ^b^Adjusted linear regression predicting chronotype; †Partial F-test p-value; AA = African American.

### 3.3 Chronotype

In both unadjusted and adjusted models, sociodemographic factors were not associated with chronotype, operationalized as MST. See Table 2.

## 4 Discussion

Pregnant women who identified as Black/African American had longer sleep duration in the unadjusted model compared to their White counterparts, which is consistent with other studies (Caraballo, 2022, Bailey, 2019). It is possible that a combination of factors may be influencing these differences including occupation and environment (Malone, 2016). For example, research suggests that Black individuals are more likely to have occupations that promote delayed sleep cycles (e.g., shift work), less flexible and/or predictable work schedules, greater job-related stress, and live in urban environments (Ertel et al., 2011, Golden, 2001, Jackson, 2013, Presser, 2016). This study’s findings that sleep duration is the longest among Black/African American respondents, at 9 h and 6 min (unadjusted), indicates an average sleep duration just above the recommended sleep duration of 7–9 h per night for all adults (Ross, 2020). Sleep recommendations specific to pregnant women do not currently exist, and such recommendations might help to further identify whether discrepancies such as the one described here (roughly 8 h for White respondents vs 9 h for Black/African American respondents) is a clinically meaningful difference.

Unlike sleep duration, we did not find significant differences in chronotype by race/ethnicity. However, studies suggest that delayed sleep cycles may be more harmful among Black/African American mothers compared to White mothers (Eastman, 2012, Smith, 2009). Typically, later chronotypes have been associated with negative health markers (e.g., elevated cardiometabolic risk (Baldanzi, 2022), which are also more prevalent among Black/African American women of childbearing age (Kazemi, 2021, Kalinowski et al., 2019). Thus, it is surprising that racial/ethnic differences in chronotype were not identified in our sample. Future research should consider the potential impact of social jetlag, or the misalignment of biological preferences with social/occupational driven sleep-wake timing, on maternal-child health outcomes. It is also recommended that future research attempt to identify potential socioeconomic factors (e.g., occupational schedule, urban/rural living environment) that may differentially impact sleep parameters among Black/African American mothers during pregnancy.

The present study identified annual household income and age as factors associated with sleep duration. Previous literature has described a relationship between low income and long sleep duration (Lallukka, 2012) In our analyses, we identified high income earners (≥100,000 USD) are those with the shortest sleep duration, roughly 8 h. This may indicate that pregnant mothers in high income earning households may, themselves, be employed in positions that demand long hours and/or produce additional stress, impacting sleep onset and/or total duration. Similarly, our adjusted findings suggest that pregnant women ages 25–34 years old are getting above recommended levels of sleep: almost 10 h (when the recommendation for adults is 7–9 h (Ross, 2020). It is unknown if or how getting more sleep than recommended for adults may negatively impact the health of pregnant women and their developing fetus, though one case control study has suggested sleeping greater than 9 h per night may be associated with stillbirth (O'Brien, 2019). Conversely, our finding may indicate that pregnant women need more sleep than the average adult, and that only those 25–34 years old are able to achieve the extra hours of sleep needed. Finally, while there was a significant association between annual household income and sleep duration in both unadjusted and adjusted models, the association between age and sleep duration only became significant after adjustment for other covariables. This might be a result of confounding by income, a factor associated with age that is not accounted for in the unadjusted model.

Findings from this study can be used to inform future studies of sleep parameters among pregnant women. In particular, future research should test the association of sleep parameters with maternal-child health outcomes of interest (e.g., gestational diabetes, low birth weight) and explore potential mediation pathways whereby sleep parameters mediate the relationships between demographic risk factors and negative health outcomes. Such studies can be used to inform the provision of targeted sleep counseling and/or behavioral interventions early in pregnancy coupled with recommendations for moderate physical activity, which can aid in sleep quality (Wang and Boros, 2019). Additionally, this research provides evidence for a need to identify sleep guidelines specific to pregnant women. Current guidelines exist for children and adults only (Ross, 2020, Tremblay, 2016). Evidence-based sleep guidelines for pregnant women are needed to inform targets for behavioral interventions in this population.

### 4.1 Limitations

Several limitations exist. The analytic sample was derived from a convenience sample of currently pregnant women in the US, and respondents self-selected to participate in the survey. The sample was relatively affluent and educated, reducing the generalizability of our results to more diverse samples. The survey was a cross-sectional, single time-point study that did not measure changes in sleep parameters overtime, nor did it incorporate a measure of the pregnancy trimester. Future studies should examine longitudinal changes during pregnancy, particularly over the different trimesters of pregnancy, which may be associated with distinct sleep patterns. The outcome measures for this analysis, sleep duration and chronotype, were asked about late in the survey, which may have contributed to missing data; it is possible that the missing data could have impacted the findings. Furthermore, not all potential covariates were available for inclusion in the models. Finally, due to the nature of data collection, sleep duration and chronotype were derived from the µMCTQ, a validated self-report measure (Centiment, xxxx). Future studies would benefit from using actigraphy to derive objective measures of sleep onset and waking.

## 5 Conclusion

Sleep duration was associated with race (unadjusted only), age (adjusted only), and income among currently pregnant women. Examining sociodemographic differences in sleep duration and chronotype among currently pregnant women can help elucidate factors associated with poor sleep, potentially leading to negative health outcomes for both woman and child. Future studies should examine the potential causal impacts of sociodemographic factors identified in this study as being associated with sleep parameters. Understanding the impact of these factors on sleep during pregnancy can inform behavioral programs to promote sleep hygiene during pregnancy.

## 6 Funding credit

This publication was made possible by the George Washington University Center of Excellence in Maternal and Child Health under Grant No T76MC35370 from the Health Resources & Services Administration (HRSA) Maternal and Child Health Bureau. Its contents are solely the responsibility of the authors and do not necessarily represent the official views of the Health Resources & Services Administration (HRSA) Maternal and Child Health Bureau.

## 7 Human subjects approval statement

This study was approved by the George Washington University Institutional Review Board (IRB).

## CRediT authorship contribution statement

**Elizabeth M. Cassidy:** Conceptualization, Data curation, Formal analysis, Investigation, Methodology, Software, Visualization, Writing – original draft, Writing – review & editing. **Caitlin P. Bailey:** Conceptualization, Data curation, Formal analysis, Investigation, Methodology, Project administration, Resources, Software, Supervision, Validation, Visualization, Writing – original draft, Writing – review & editing. **Melissa A. Napolitano:** Conceptualization, Data curation, Investigation, Methodology, Resources, Supervision, Writing – review & editing. **Amita N. Vyas:** Conceptualization, Data curation, Funding acquisition, Investigation, Methodology, Resources, Supervision, Writing – review & editing.

## Declaration of Competing Interest

The authors declare that they have no known competing financial interests or personal relationships that could have appeared to influence the work reported in this paper.

## Data Availability

The authors are unable or have chosen not to specify which data has been used.
